# Introduction to the Special Issue on Structural Approaches to Youth Violence Prevention: Addressing Racism and Discrimination

**DOI:** 10.1007/s11121-026-01951-x

**Published:** 2026-07-04

**Authors:** Krista R. Mehari, Catherine P. Bradshaw

**Affiliations:** 1 Charles Stewart Mott Department of Public Health, Michigan State University, Flint, MI USA; 2 School of Education and Human Development, University of Virginia, Charlottesville, VA USA

**Keywords:** Youth violence, Systems, Root causes, Health disparities, Equity

## Abstract

Youth violence is a significant public health concern, for which a number of preventive approaches have demonstrated promise in reducing the overall harm and associated effects. Yet relatively few of those approaches have explicitly addressed the role of structural racism and discrimination, which is a potential root cause of youth violence. This paper introduces a special issue of *Prevention Science*, “Structural Approaches to Youth Violence Prevention: Addressing Racism and Discrimination,” which includes a collection of original papers from multiple disciplines that outline promising approaches for advancing prevention science in this important and timely direction. The overarching goal of the special issue is to provide insights related to youth violence prevention by directly addressing racism and discrimination at the structural level. The papers in this issue include systematic reviews, proposed approaches to policy and practice, intervention development and process, and evaluation research. After identifying common themes across the papers, we conclude with some future directions for research on structural interventions for youth violence prevention.

Youth violence is a major health disparity in the USA, with youth who are members of marginalized groups disproportionately experiencing the burden of violence. Although the disparity by race and ethnicity is commonly identified, with Black and Brown youth much more likely to be victimized by violence (Catalano, 2006; Gage et al., 2021), other groups also experience an undue burden of violence. For example, youth are significantly more likely to be victimized by violence when they are sexual or gender minorities (Garthe et al., 2021; Hazelwood, 2023; Lowry et al., 2020), when they have a disability (Gage et al., 2021; Jones et al., 2012; Yun et al., 2015), when they have observable physical characteristics such as obesity (Farhat et al., 2010; Waasdorp et al., 2018), when their families have lower income (Crouch et al., 2000), and when English is not their first language (Peguero, 2008). Despite this pattern in which marginalized groups are much more vulnerable to violence, evidence-based interventions to prevent youth violence by addressing structural inequities remain scarce in the published literature. This special issue of *Prevention Science* highlights the latest advances in the conceptualization, development, testing, and dissemination of community-engaged, multilevel youth violence prevention interventions that address racism/discrimination and other structural and social determinants. This special issue brings together scholars funded by the National Institute of Minority Health and Health Disparities initiative on youth violence prevention (i.e., RFA-MD-18–005), as well as other scholars in the field of youth violence prevention, to describe their work on preventive intervention development, implementation, and evaluation through an equity lens (Boyd et al., 2023). To date, the violence prevention field has centered its efforts on individual-level, youth-focused violence prevention strategies. Although progress has been made in some areas of youth violence prevention (Bottiani et al., 2021; Fazel et al., 2024), there is a strong need for violence prevention researchers and practitioners to identify and address the structural factors that cause, maintain, or perpetuate youth violence. This special issue highlights emerging work in this area to stimulate discourse and creativity in intervention development, implementation, and dissemination with the overarching goal of moving toward a society in which all young people have an equal opportunity to be safe.

## Special Issue Overview

### Systematic Reviews

The first set of articles in this special issue systematically reviews the literature for research on the intersection of youth violence prevention and structural interventions that may reduce race-based disparities. Weise and colleagues (2026) searched the literature for youth violence prevention studies that included structural-related keywords, including discrimination and racism, and found that body of research is in its infancy. Where possible, they also conducted an intervention component analysis. They concluded that several of the studies did include multicomponent interventions, but relatively few directly tested the change mechanisms. They also identified future directions for research on structural approaches to youth violence prevention, including the need for intervention mapping, the identification of active ingredients, documentation of implementation quality, and a greater focus on “upstream drivers” in preventive interventions to reduce youth violence.

Joseph and Jay (2025) reviewed summer youth employment programs as a strategy to reduce not only youth violence but also to improve community safety and cohesion. Their review found that most outcomes were measured at the individual level and demonstrated reductions in youth violence; at the interpersonal and community levels, outcomes included increased prosocial networks. Joseph and Jay contended that because summer youth employment programs are widespread and sustainable, they are a strong avenue to address societal determinants of health. However, they cautioned that inequitable implementation without community empowerment could increase inequities and that more research on implementation and societal-level impacts are needed.

Scott and colleagues (2025) reviewed interventions that addressed racism in school disciplinary practices, given previous research showing that attempting to reduce exclusionary discipline without specifically addressing disproportionate discipline either maintained or exacerbated disparities. They found that programs that include culturally responsive practices are promising to reduce disproportionate discipline. However, implementation and sustainability may present significant challenges for these and other such prevention programs, and thus, sufficient investments in implementation systems and infrastructure should be directly addressed.

### Proposed Approaches to Policy and Practice

The second set of articles describes proposed changes in policy and practice. Jahangir and colleagues (2026) argued that economic security policies are a societal-level approach that has strong potential to reduce youth violence by addressing poverty and economic opportunity. A major barrier to the success of economic security policies is the phenomenon of marginalization-related diminished returns, in which programs have smaller benefits for Black than White Americans. The authors proposed a strategy to counteract these diminished returns through community engagement in evaluating policies based on their implementation, not just their presence. They described a process in which an evaluation partnership with a community action board led to actionable policy implementation changes that would likely move toward equity in who benefits from economic security policies.

Gastineau and colleagues (2026) highlighted disparities in hospitals’ standard practice for treating pediatric firearm injuries compared to chronic illnesses. They pointed to provider biases, structural discrimination, and society’s framing of firearm injuries as a criminal issue rather than a public health issue as major factors affecting the care for Black and other minoritized youth. They identified structural issues that present barriers to the adequate postinjury support of youth who have sustained a firearm injury. To counteract these inequities, Gastineau et al. provided recommendations for hospital standards in pediatric firearm injury care.

### Intervention Development and Process

The third set of articles describes processes used by teams to build, adapt, or evaluate interventions that address structural racism or discrimination as root causes of youth violence. These approaches all included engagement from members of impacted communities, ranging from community-led and community-driven work, including youth participatory action research (YPAR), to community-informed work. The papers by Lugo and colleagues (2025) and Yusef and colleagues (2025) both described YPAR approaches. In New Orleans, the Enrichment to Empowerment program empowered youth to conduct research and take collective public action to advocate for city-level change (Lugo et al., 2025). The authors acknowledged a tension between the need for flexibility and power-sharing and the external deadlines from agencies and institutions. In Iowa, a university–community partnership engaged in YPAR to adapt a sexual violence prevention logic model to be culturally and contextually grounded for maximum impact among Asian American and Pacific Islander youth (Yusef et al., 2025). The authors argued that a YPAR approach itself fights against structural discrimination through the redistribution of power.

Another approach highlighted in this special issue was the use of theories of change and logic models to guide intervention implementation by communities for communities (Fields et al., 2026; Jones et al., 2026; Thompson et al., 2025). Fields and colleagues (2026) described an intervention mapping approach that was used to adapt a youth violence prevention, youth-focused curriculum developed in the Global South for use in Pittsburgh through a university–community partnership. Adaptations included using an antiracist framework, expanding the emphasis on youth activities, and integrating inclusiveness across sexual orientation, gender identity, and intersectional identities. Although a youth-focused intervention, it was conceptualized as structural by empowering youth to critique and engage in activism to address structural root causes of youth violence. Jones et al. (2026) used qualitative interviews with leaders and staff in Washington State’s Community Violence Interrupter programs to explore which mentorship components serve as active ingredients to reduce youth violence. Mentorship was conceptualized as a structural intervention that impacted resources, access, and social networks. Thompson and colleagues (2025) described the efforts of a community coalition of 40 community partners, ThrYve (Together Helping Reduce Youth Violence for Equity), which used the Institute of Medicine Model for Collaborative Public Health Action to assess, plan, and enact community-level changes to reduce youth violence. Community partners selected and led 10 creative change levers, which were implemented across social–ecological levels. The authors noted that intervention implementation was steady across 4 years, suggesting sustainable change, and that the focus of interventions shifted from individual (youth)-focused interventions to structural interventions over time, highlighting the amount of time, effort, and planning required to implement structural interventions.

Two papers focused on the development of new, community-informed interventions to address structural discrimination. Both Edberg and colleagues (2026) and Smith Lee and colleagues (2025) pointed to one root cause of youth violence as the cultural narratives that restrict and diminish the perception of the capacities, identities, and dignity of Black youth in general and Black boys and young men in particular. To address this, Edberg and colleagues (2026) worked with a community steering community and youth advisory committee to create Run It Up, a narrative reframing intervention, the goal of which was to expand the range of possible, prosocial selves and equip youth to pursue those possible selves. Guided by cultural persona theory, ecological positive youth development theory, and branding theory, they developed storylines of personas that were prosocial and associated with perceptions of power, respect, money, and attractiveness, which they launched through a media campaign. They also integrated a youth-focused training and curriculum that provided resources for youth to pursue those possible selves. In a similar attempt to change harmful narratives, Smith Lee and colleagues (2025) developed In All Ways Human in Baltimore, MD, to change racialized narratives of firearm violence (e.g., self-inflicted, due to criminal natures, irrelevant to the nation as a whole) driven by the historical and current dehumanization of Black individuals. In their intervention, In All Ways Human, the authors used interviews and portraits to push back against stereotypes by bringing to the forefront the deep humanity of Black boys and men. They measured effectiveness through experimental testing as well as through indicators of spread, uptake, and resistance.

### Evaluation Research

The majority of the papers in this special issue provided development or process descriptions alongside proposed evaluations, rather than describing completed evaluations of the impact of structural interventions on youth violence. This pattern underscores that research evaluating the impact of interventions targeting structural racism and discrimination on youth violence is in its infancy. Bottiani and colleagues (2026), however, conducted a school-level randomized trial examining whether supplementing social–emotional learning curricula with R-CITY (Reducing Racism and Violence through Collaborative Intervention with Teachers and Youth) increased the impact on youth violence. R-CITY was developed through a research–practice partnership and supplements social and emotional learning with six equity-focused lessons as well as with teacher coaching and implementation support. They found evidence that R-CITY may increase the impact on youth aggression, which suggests that programs addressing structural racism and discrimination do provide additional value in reducing youth violence.

## Conclusions and Implications

Taken together, this collection of innovative studies is designed to inspire researchers, practitioners, and communities to explore a range of possibilities in developing, implementing, and evaluating structural interventions that address racism or discrimination as root causes of youth violence. Although this work is in its infancy, with most studies still ongoing, the teams whose work is shared in this issue provided important information about their partnerships, theories of change, processes, and future directions. For example, all the teams highlighted the importance of academic–community partnerships, in which the academic partners could provide resources (including coordination, money, effort, and field-specific skills and knowledge) and the community partners could provide resources (including trusted connections to impacted groups, space, networks, effort, local knowledge, and applied experience). Furthermore, shifting knowledge capital and decision-making power from the hands of academics to the hands of impacted communities was identified as a key piece of dismantling structural discrimination in academic research. Dismantling structural discrimination in research is an important end in itself; for youth violence prevention specifically, equity in the conduct of research may enable equity in research outcomes.

The research highlighted in this collection builds on a robust body of youth violence prevention literature and presents structural interventions as the logical, important next step in maximizing the equity and effectiveness of youth violence prevention strategies. Although federal funding priorities are uncertain, prevention researchers must continue to explore innovative ways to move forward in creating safety for all youth. These studies reflect a deep and growing commitment to health equity in prevention science research and practice (Boyd et al., 2023) and serve as a model for future studies of this kind to move the needle on structural approaches to youth violence prevention.

## Data Availability

No new data were created or analyzed in this study. Data sharing is not applicable to this article.
